# Early regression of hepatic steatosis after bariatric surgery assessed by ultrasound including attenuation imaging

**DOI:** 10.1186/s12880-026-02882-3

**Published:** 2026-10-22

**Authors:** Tatjana Hoffmann, Daniel Wulff, Sabrina Groß, Bariya Bajwa, Gunnar Blumenstock, André Mihaljevic, Nisar Malek, Lisa Minn

**Affiliations:** 1 Department of Internal Medicine I, University Hospital Tübingen, Tübingen, Germany; 2 Department of General, Visceral, and Transplant Surgery, University Hospital Tübingen, Tübingen, Germany; 3 Department of Clinical Epidemiology and Biostatistics, Eberhard Karls Universität Tübingen, Tübingen, Germany

**Keywords:** Ultrasound, Attenuation imaging, Quantification, Hepatic steatosis, Bariatric surgery, MASLD, BMI

## Abstract

**Background:**

Metabolic dysfunction-associated steatotic liver disease (MASLD) is associated with increased liver-related morbidity. Bariatric surgery provides substantial weight loss and metabolic improvement, yet data on ultrasound (US)-based monitoring remain limited. This study evaluated perioperative changes in hepatic steatosis using semiquantitative B-mode US and Attenuation Imaging (ATI), a quantitative US technique for fat quantification.

**Methods:**

In this prospective single-center study, patients undergoing bariatric surgery between February 2024 and July 2025 were included. B-mode US and ATI were performed preoperatively and at 3 and/or 6 months postoperatively. For overall analysis, the earliest available postoperative examination was defined as the early follow-up. Hepatic steatosis was assessed using B-mode US grading (S0–S3) and ATI grading (aS0–aS3). Body mass index (BMI), liver size, and attenuation coefficient (AC) values were recorded.

**Results:**

89 patients were included (mean age 42.8 years; 72% female). US examinations were performed at 3 months in 75.2% of patients and at 6 months in 51.7%. Of all patients, 26.9% attended both follow-up visits. At baseline, B-mode and ATI were strongly correlated (τ_b = 0.73, *p* < 0.001). At early follow-up, BMI decreased in all patients (*p* < 0.001). Grading of hepatic steatosis improved in 56.2% of patients by B-mode (*p* < 0.001) and in 68.5% by ATI (*p* < 0.001), while absolute ATI values decreased in 94.4%. Liver size decreased in 94.4%. In patients with both follow-up visits, largest reductions in ATI and liver size were observed at 3 months. Changes in B-mode and ATI grading were strongly correlated (τ_c = 0.65, *p* < 0.001).

**Conclusions:**

Regression of hepatic steatosis occurs early after bariatric surgery. ATI complements B-mode US by providing quantitative longitudinal assessment.

## Background

Metabolic dysfunction-associated steatotic liver disease (MASLD) is an increasing global health concern, affecting 25–30% of the general population and up to 70–90% of severely obese individuals [1]. While this increase may not have immediate clinical significance, patients with MASLD are at risk of developing metabolic dysfunction-associated steatohepatitis (MASH) and associated complications [2].

Hepatic steatosis is more dynamic than fibrosis, responding to weight changes induced by diet, exercise, pharmacotherapy, or bariatric surgery [3]. The primary treatment of MASLD is lifestyle modification and weight loss. Vilar-Gomez et al. demonstrated that weight loss ≥ 10% was associated with significant resolution of MASH in 90% and regression of fibrosis in 45% of patients [4]. Bariatric surgery is recommended for patients with BMI ≥ 40 kg/m^2^ or ≥ 35 kg/m^2^ with comorbidities and induces substantial and sustained weight loss [5].

A biopsy-controlled meta-analysis demonstrated that bariatric surgery can lead to complete resolution of MASLD and lower MASLD activity scores; however, follow-up examinations were performed relatively late (median 15 months) [6]. Likewise, a systematic review of liver biochemistry following bariatric surgery also reported significant reductions in hepatic steatosis and inflammation, whereas the effect on fibrosis remains controversial [7]. In a prospective study, bariatric surgery resulted in marked reductions in liver fat content by MRI-derived proton density fat fraction (MRI-PDFF), liver volume, and BMI within 6 months, with 83.7% of patients achieving resolution of hepatic steatosis [8]. These findings are supported by a systematic review of 12 prospective studies including 613 patients, which demonstrated significant reduction in MRI-PDFF–derived intrahepatic fat content after bariatric surgery; greater weight loss was consistently associated with larger reductions in liver fat, with several studies also reporting resolution of steatosis and MASH [9].

Liver biopsy and magnetic resonance imaging (MRI) allow accurate quantification of hepatic steatosis but are limited by invasiveness, cost, and restricted availability. Conventional B-mode ultrasound (US) is widely used for semiquantitative assessment of hepatic fat, graded from 0 (absent) to 3 (severe) [10]. A meta-analysis demonstrated diagnostic accuracy comparable to CT or MRI for moderate-to-severe hepatic steatosis when histology was used as the reference standard [11]. Despite advances in US technologies today, qualitative B-mode US remains effective for the detection of hepatic steatosis [12]. However, B-mode US is less sensitive for mild steatosis and is associated with significant interobserver variability [11].

Recently, quantitative US methods have emerged as promising noninvasive biomarkers for hepatic steatosis [13]. One such technique, attenuation imaging (ATI), measures US beam attenuation and calculates an attenuation coefficient (AC) as a surrogate marker for liver fat. Steatotic tissue attenuates US beams more strongly than normal liver tissue, resulting in higher AC values. In the present study, ATI implemented on a Canon Medical Systems platform was used. Previous studies using this platform have reported that ATI showed higher diagnostic accuracy than the controlled attenuation parameter (CAP) across all steatosis grades (S1–S3), using MRI proton density fat fraction (MRI-PDFF) as the reference standard [14]. Furthermore, ATI has demonstrated a strong correlation with both MRI-PDFF and histological assessment of hepatic steatosis [15, 16]. In addition, several other AC-based techniques have been developed by different US manufacturers, including US attenuation technology (USAT; Mindray), US-guided attenuation parameter (UGAP; GE HealthCare), and US-derived fat fraction (UDFF; Siemens Healthineers). Although these techniques require vendor-specific cut-off values because of differences in acquisition algorithms and US platforms, they have consistently demonstrated good diagnostic accuracy for hepatic steatosis when validated against MRI-PDFF [17–19].

Reproducibility studies have reported good-to-excellent intra- and interobserver agreement for AC-based methods, with intraclass correlation coefficients consistently exceeding 0.80 [3]. More recent investigations have further confirmed excellent interobserver reproducibility of attenuation measurements [20].

Published data in patients with MASLD are particularly promising [21]. ATI has demonstrated diagnostic performance comparable to, and in some studies superior to, the CAP while offering practical advantages, including integration into conventional B-mode US systems and improved feasibility in obese patients [14, 22]. Reflecting the growing evidence supporting quantitative ultrasound techniques, the World Federation for Ultrasound in Medicine and Biology (WFUMB) recently published updated recommendations for US-based liver fat quantification [3].

Recent studies suggest that quantitative assessment using ATI identifies more patients with hepatic steatosis than qualitative B-mode US, particularly in patients with mild steatosis [23]. To improve diagnostic consistency, a standardized ATI grading system has recently been proposed. In a meta-analysis including 1,374 patients with MRI or biopsy as reference standards, ATI values correlated significantly with steatosis grades [24]. Based on these findings, ATI grading was defined as follows: aS0 (ATI ≤ 0.66), aS1 (0.66 < ATI ≤0.73), aS2 (0.73 < ATI ≤0.81), and aS3 (ATI ≥ 0.81) [24].

To our knowledge, there is currently no data on early postoperative changes in hepatic steatosis assessed by B-mode US and ATI in patients undergoing bariatric surgery. Given that US is noninvasive, rapid, cost-effective, and widely available, its role in longitudinal postoperative monitoring warrants further investigation.

The primary objective of this study was to assess whether substantial postoperative weight loss results in detectable reduction in hepatic steatosis at early follow-up. The secondary objective was to evaluate the additional value of ATI for longitudinal liver fat assessment in this patient population.

## Methods

### Ethical approval

The study was approved by the Ethics Committee of the University of Tübingen (approval number 042/2024BO2), and all participants provided written informed consent. The study was conducted according to the declaration of Helsinki.

### Study design

This prospective single-center study included 89 patients undergoing first-time bariatric surgery between February 2024 and July 2025. Of these, 65 underwent sleeve gastrectomy, 19 underwent Roux-en-Y gastric bypass, and 5 underwent Omega-loop gastric bypass. Inclusion criteria were age > 18 years, ability to provide consent, and comprehension of German language. Exclusion criteria were chronic liver disease, insufficient compliance, or participation in other studies.

### Data collection

Baseline examination, including B-mode US and ATI, was performed preoperatively. Follow-up examinations were scheduled at 3 and/or 6 months postoperatively. For overall analysis, the earliest available follow-up was defined as early follow-up. Demographic and anthropometric data, including age, sex, BMI, liver size, and mean AC, were recorded at each examination.

### US imaging and ATI measurements

US examinations were performed by two DEGUM-certified gastroenterologists using an Aplio i800 system (Canon Medical Systems) equipped with a 1–8 MHz convex abdominal transducer (i8C×1), according to current WFUMB recommendations [3]. Baseline and follow-up examinations were performed by the same examiner. Patients were instructed to fast for at least 3 hours before the examination.

Using B-mode US, hepatic steatosis was graded according to a widely used four-point scale [25]: score 0 indicated normal hepatic echogenicity; score 1 indicated mildly increased echogenicity with preserved visualization of the diaphragm and portal vein borders; score 2 indicated moderate echogenicity with impaired visualization of periportal structures; and score 3 indicated marked echogenicity with poor visualization of periportal structures and the posterior right hepatic lobe.

**Fig. 1:**
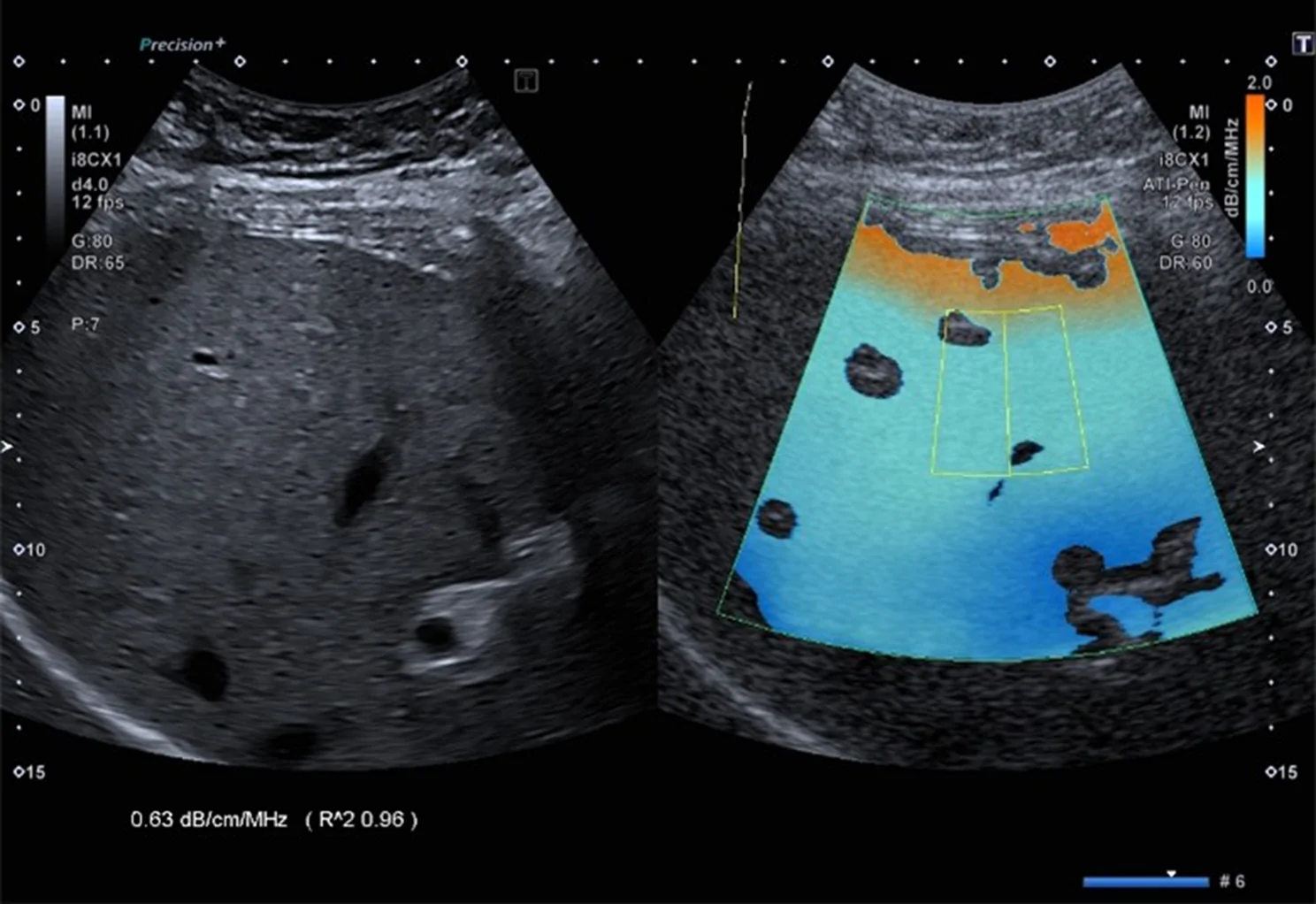
Measurement of hepatic steatosis using attenuation imaging (ATI)

For ATI assessment, as recommended by Ferraioli et al. [26], all patients were examined in the supine position, with 3–5 seconds of breath holding. After optimization of the B-mode image, ATI measurements were acquired from the right hepatic lobe through an intercostal approach using a 3-cm ROI placed with its top 2 cm below the liver capsule in homogeneous liver parenchyma. Only measurements with an attenuation fit correlation coefficient (R^2^) >0.80 were accepted. Five valid measurements were taken from the parenchyma, excluding vascular structures and gall bladder. Measurements were performed in real time using a color-coded overlay on the B-mode image, as shown in Fig. 1. AC values (dB/cm/MHz) were calculated while accounting for beam profile, gain settings, time gain compensation, and signal compounding. Hepatic steatosis was additionally categorized using the ATI four-scale classification proposed by Hobeika et al. [24], with cutoff values of 0.66, 0.73, and 0.81 dB/cm/MHz.

### Statistical analysis

Continuous data on BMI, liver size, and ATI values were measured at baseline and at follow-up (3 and/or 6 months postoperatively), and are presented using box and whisker plots. B-mode US as well as ATI were classified on a four-graded ordinal scale. Changes from baseline for repeated measurements were examined using the two-sided paired t-test or by repeated-measures ANOVA. For all tests, the level of statistical significance was predetermined at *p* ≤ 0.05. Kendall’s tau coefficient was used as measure of ordinal association with a correction for the number of ties (Tau-b) and an additional adjustment for table size (Stuart-Kendall Tau-c). The strength of the observed relationship was assessed, and a Kendall tau significance test was performed to check if the ordinal association is statistically different from zero. To indicate the degree of ordinal scale agreement between B-mode and ATI grading, Cohen’s quadratic weighted kappa κ_w_ is also provided with 95% confidence limits. Data analysis was performed using JMP 18.2 statistical software (JMP Statistical Discovery LLC, Cary, NC, USA).

## Results

### Baseline characteristics

Table 1 summarizes the demographic and clinical characteristics of the patients at baseline. All included individuals were classified as having class III obesity, with a mean BMI value of 49.4 kg/m^2^ (±7.3). The mean age was 42.8 years, and 71.9% (64/89) were women.

**Table 1:** Demographic and clinical patient characteristics at baseline

Number (n)	89
Male/Female	25/64
Mean age (range)	42.8 yrs (19–73)
Mean BMI (SD)	49.4 kg/m^2^ (7.3)
Grade of steatosis based on B-Mode	
S0	10 (11.2%)
S1	45 (50.6%)
S2	26 (29.2%)
S3	8 (9.0%)
Mean ATI absolute value (range)	0.74 dB/cm/MHz (0.58–0.96)
Grade of steatosis based on ATI	
aS0	19 (21.3%)
aS1	31 (34.8%)
aS2	19 (21.3%)
aS3	20 (22.5%)
Mean Liver Size (range)	17,4 cm (11,5–23)

Regarding ATI measurement, the mean AC was 0.74 dB/cm/MHz. As expected, the majority of patients (88.8%, 79/89) exhibited signs of hepatic steatosis on baseline B-mode US. Among these 50.6% (45/89) were classified as S1, 29.2% (26/89) as S2, and 9.0% (8/89) as S3.

According to the four-scale classification of Hobeika et al. 78.7% (70/89) showed signs of hepatic steatosis in ATI. Of these, 34.8% (31/89) were classified as aS1, 21.3% (19/89) as aS2, and 22.5% (20/89) as aS3.

### Changes at follow-up

Follow-up US examinations were performed at 3 months in 75.2% (67/89) of patients and at 6 months in 51.7% (46/89). Among the cohort, 26.9% (24/89) attended both follow-up visits, 48.3% (43/89) only the 3-month follow-up, and 24.7% (22/89) only the 6-month follow-up.

### Body mass index (BMI)

**Fig. 2:**
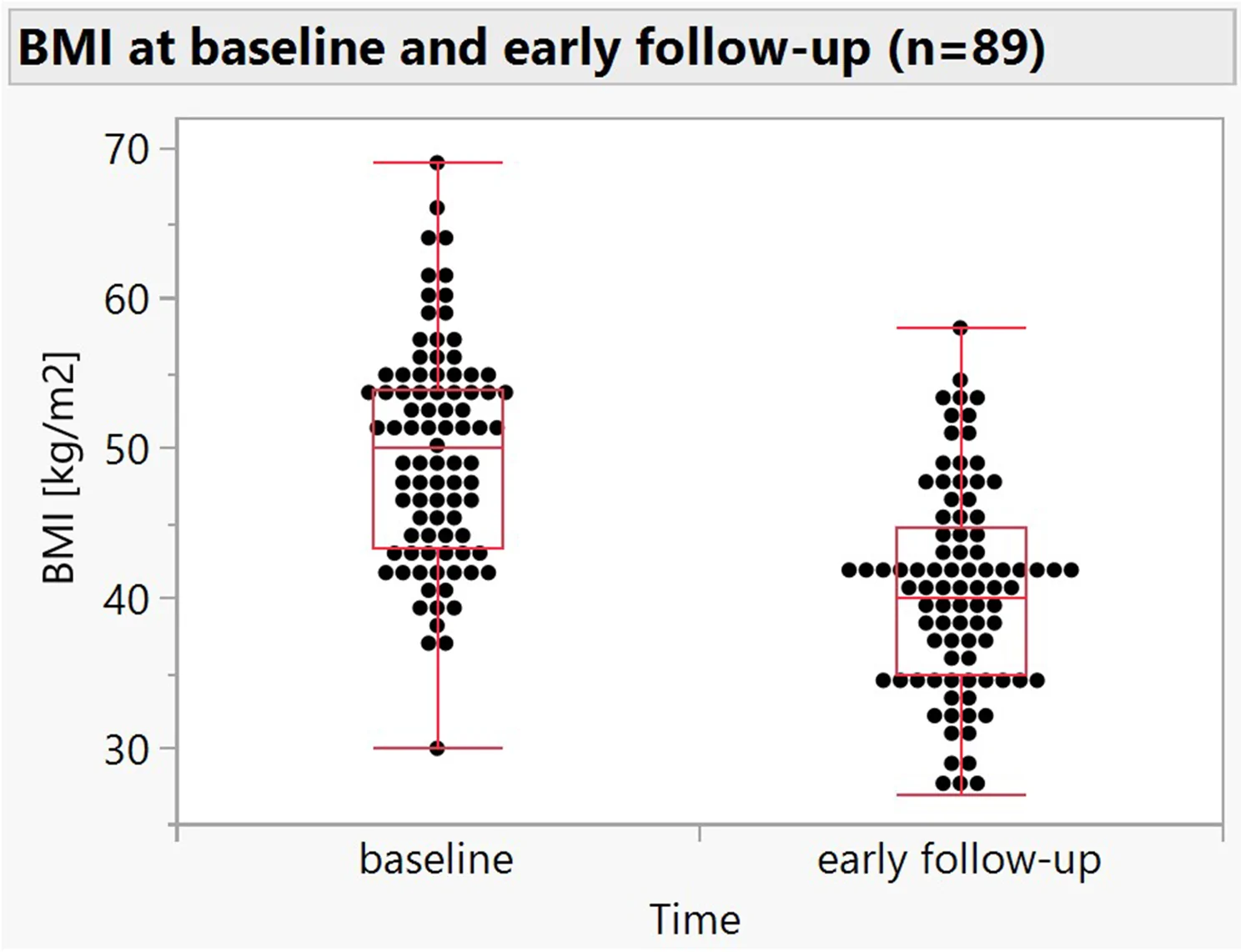
BMI-scores at baseline and early follow-up. Used statistical test: two-tailed paired t-test. Mean difference −9.2 (95%-CI [−9.9 to −8.4]), Cohen´s d = 2.68 (95%-CI [2.22 to 3.12]), *p* < 0.001

Following bariatric surgery, all patients demonstrated a significant reduction in BMI during early follow-up, as shown in Fig. 2. The mean preoperative BMI was 49.4 ± 7.3 kg/m^2^, which decreased to 40.4 ± 6.7 kg/m^2^ at early follow-up (*p* < 0.001).

**Fig. 3:**
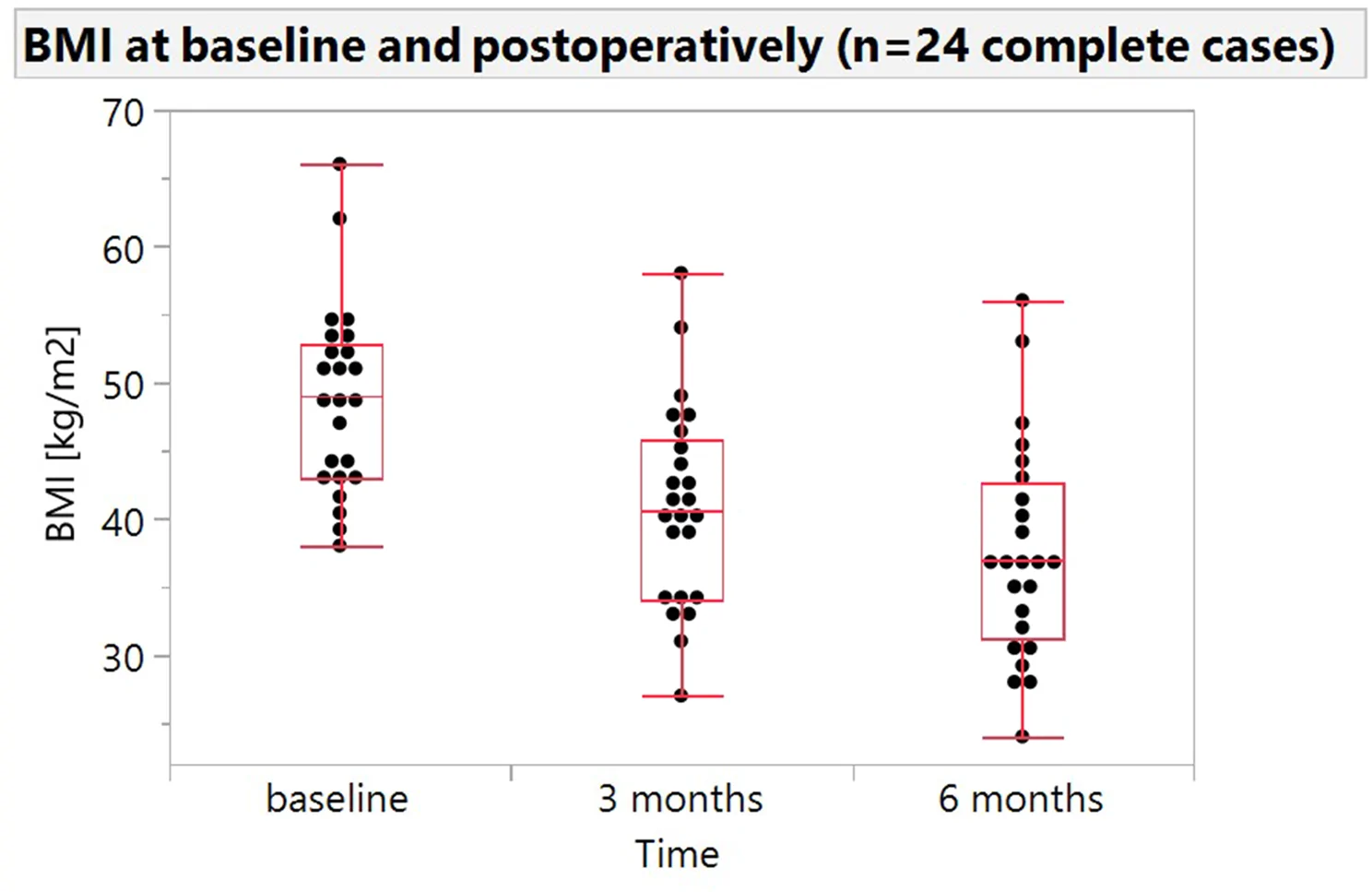
Changes in BMI scores from baseline to 3 and 6 months after bariatric surgery. Used statistical test: repeated-measures ANOVA. Baseline − 3 months: mean difference −7.8 (95%-CI [−8.7 to −6.9]), Cohen´s d = 3.84 (95%-CI [2.60 to 5.05]), *p* < 0.001. Baseline −6 months: mean difference −11.1 (95%-CI [−12.7 to −9.5]), Cohen´s d = 3.10 (95%-CI [2.07 to 4.12]), *p* < 0.001

As shown in Fig. 3, among patients who attended both follow-up visits, the decrease in BMI was more pronounced during the first 3-months. At 3 months, the mean BMI was 40.9 kg/m^2^, corresponding to a reduction of 7.8 kg/m^2^ from baseline. At 6 months, the mean BMI further decreased to 37.4 kg/m^2^, representing an additional reduction of 3.5 kg/m^2^.

### Attenuation imaging (ATI)

**Fig. 4:**
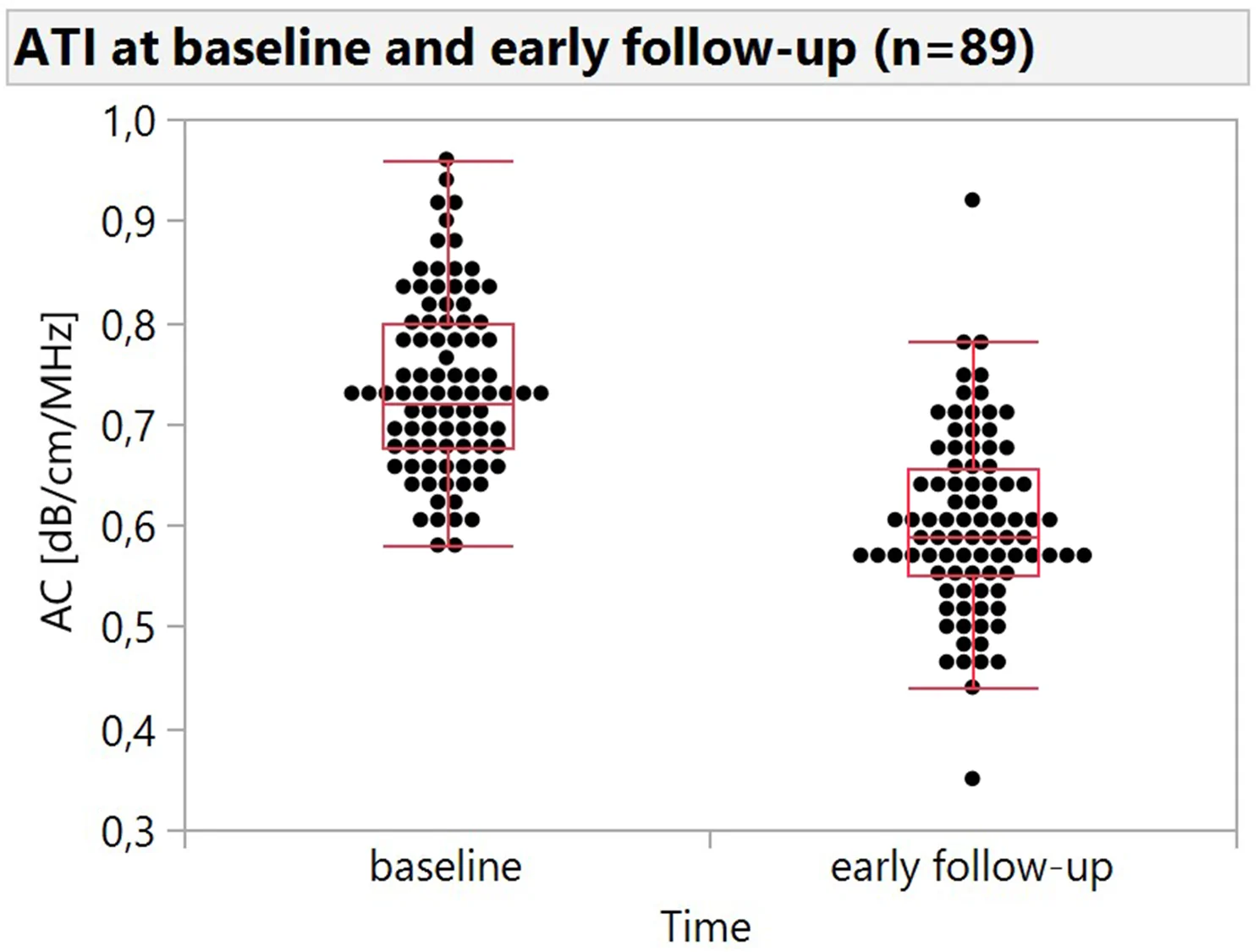
AC values at baseline and early follow-up. Used statistical test: two-tailed paired t-test. Mean difference −0.136 (95%-CI [−0.157 to − 0.115]), Cohen´s d = 1.37 (95%-CI [1.08 to 1.66]), *p* < 0.001

As shown in Fig. 4, analysis of the absolute ATI values revealed a significant decrease at early follow-up compared to baseline (*p* < 0.001). The mean preoperative ATI value was 0.73 dB/cm/MHz (range: 0.58–0.96), which decreased to 0.60 dB/cm/MHz (range: 0.35–0.92), corresponding to a reduction of 0.14 dB/cm/MHz. Overall, 94.4% of patients exhibited a decrease in ATI values. In the subgroup of patients with only a 6-month follow-up (22/89), 100% showed a reduction in absolute ATI values.

**Fig. 5:**
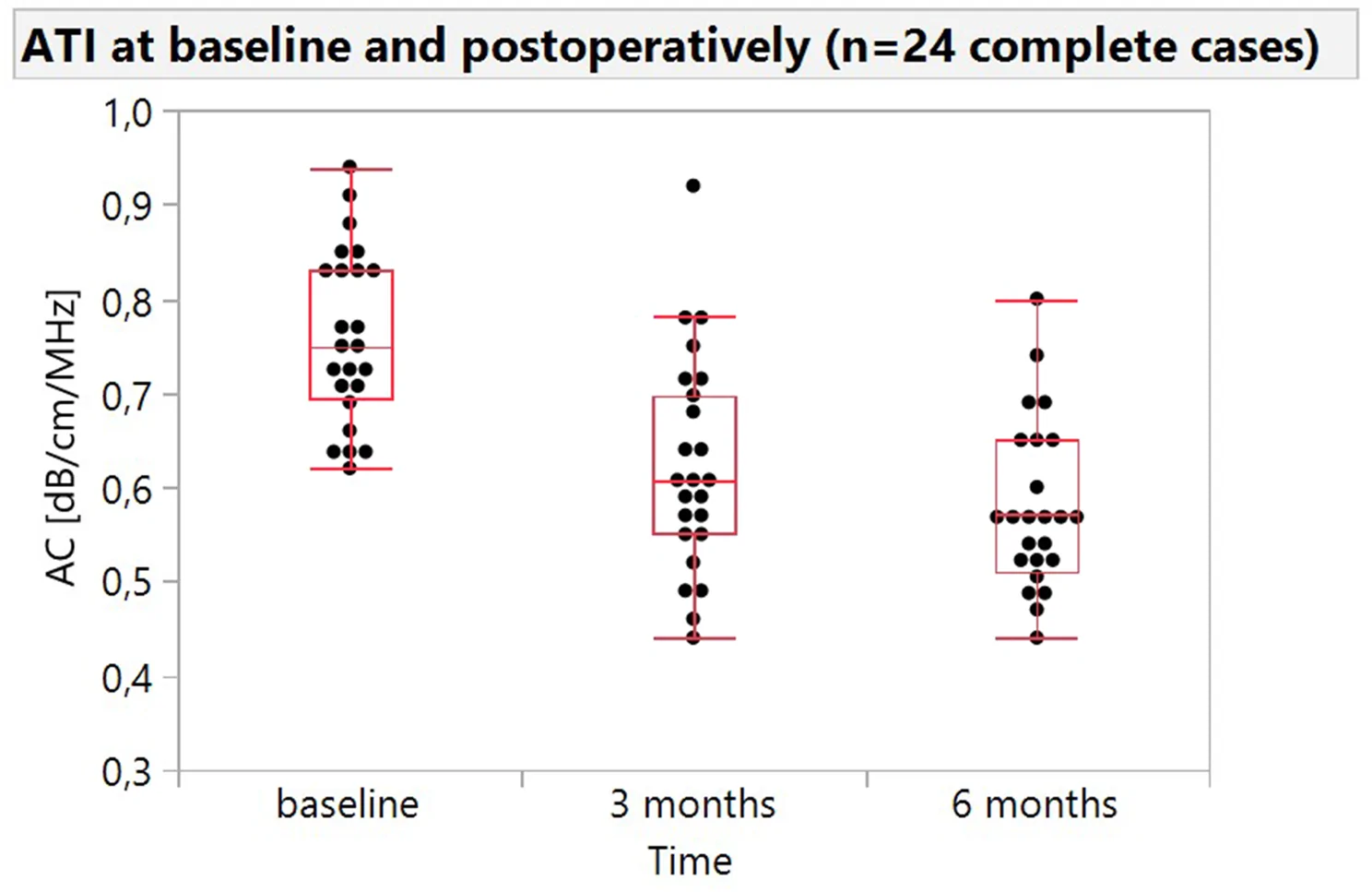
Changes in AC values from baseline to 3 and 6 months after bariatric surgery. Used statistical test: repeated-measures ANOVA. Baseline-3 months: mean diff erence −0.138 (95%-CI [−0.182 to −0.093]), Cohen´s d = 1.29 (95%-CI [0.74 to 1.83]), *p* < 0.001. Baseline-6 months: mean difference −0.182 (95%-CI [−0.224 to −0.140]), Cohen´s d = 1.28 (95%-CI [1.15 to 2.47]), *p* < 0.001

As illustrated in Fig. 5, among the 24 patients attending both follow-up visits, the reduction in ATI values was more pronounced at 3 months. At this time point, the mean ATI value was 0.61 dB/cm/MHz, corresponding to a decrease of 0.15 dB/cm/MHz from baseline. At 6 months postoperatively, the mean ATI value further decreased to 0.57 dB/cm/MHz.

### Liver size

**Fig. 6:**
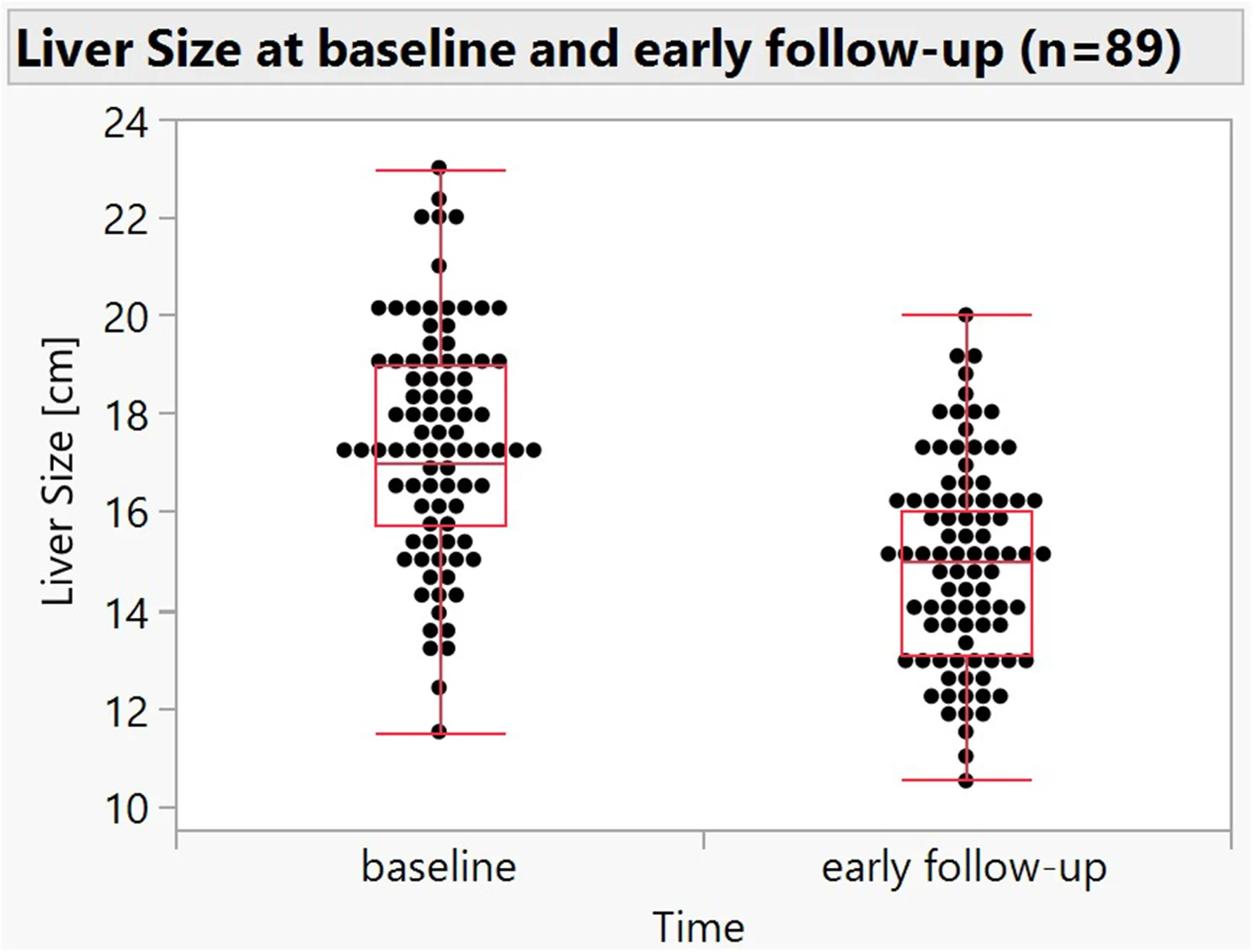
Liver size at baseline and early follow-up. Used statistical test: two-tailed paired t-test. Mean difference −2.54 (95%-CI [−2.87 to −2.21]), Cohen´s d = 1.63 (95%-CI [1.31 to 1.95]), *p* < 0.001

Analysis of liver size demonstrated a significant decrease during early follow-up, as shown in Fig. 6. The mean preoperative liver size was 17.4 cm (range: 11.5–23.0 cm), which decreased significantly at early follow-up (*p* < 0.001) to a mean of 14.8 cm (range: 10.5–20.0 cm), corresponding to an average decrease of 2.6 cm compared to baseline.

**Fig. 7:**
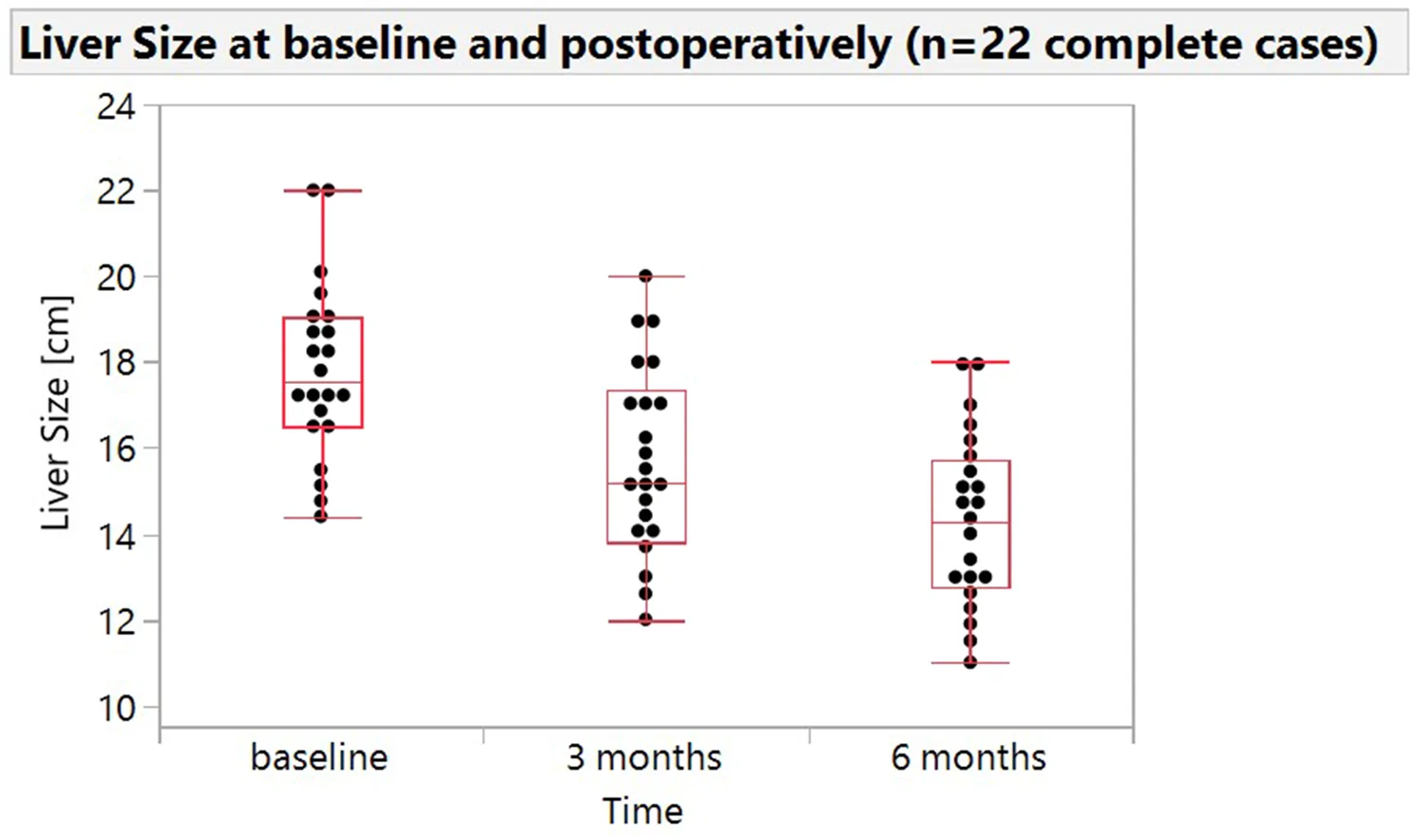
Changes in liver size from baseline to 3 and 6 months after bariatric surgery. Used statistical test: repeated-measures ANOVA. Baseline-3 months: mean difference −2.08 (95%-CI [−2.26 to −1.49]), Cohen´s d = 1.62 (95%-CI [0.95 to 2.26]), *p* < 0.001. Baseline-6 months: mean difference −3.43 (95%-CI [−4.25 to −2.61]), Cohen´s d = 1.90 (95%-CI [1.17 to 2.62]), *p* < 0.001

As demonstrated in Fig. 7, among the patients who attended both follow-up visits, liver size decreased more markedly during the first 3 months. At 3 months, the mean liver size was 15.7 cm, corresponding to a reduction of 2.1 cm from the baseline value of 17.8 cm. At 6 months postoperatively, liver size further decreased to 14.3 cm, representing an additional reduction of 1.4 cm.

### Relationship between US and ATI

At baseline, the relationship between US and ATI grading of hepatic steatosis showed a strong and statistically significant positive correlation assessed by Kendall`s Tau correlation analysis (τ_b = 0.73; 95% CI 0.66–0.81; *p* < 0.001), as shown in Table 2. The weighted kappa statistic was κ_w_ = 0.76 (95% CI: 0.68–0.84).

**Table 2:**
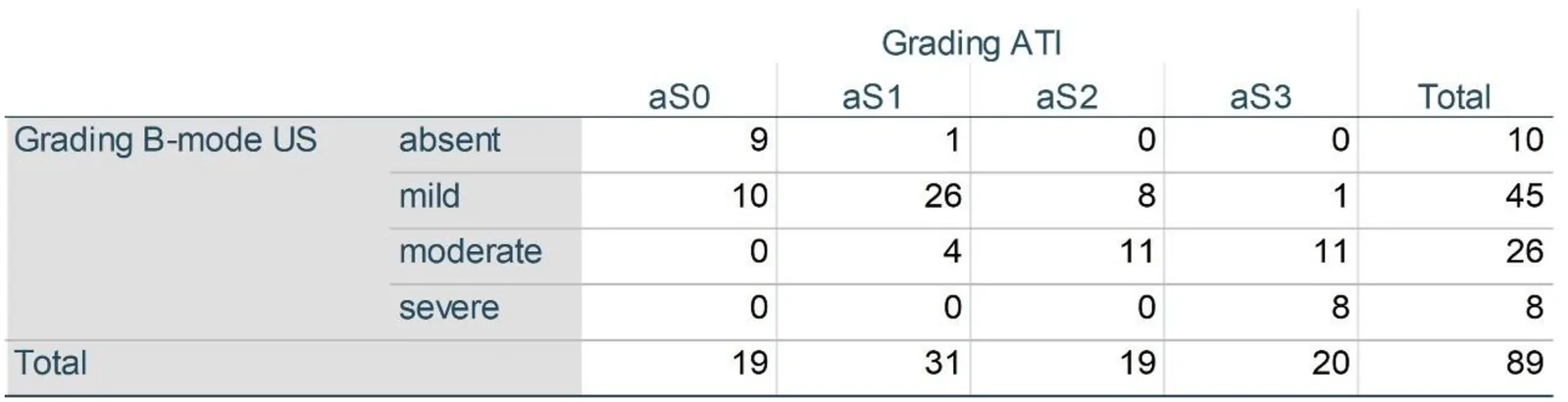
Association between B-mode US and ATI

Changes in steatosis grading from baseline to early follow-up were also strongly positively associated between B-mode US grading and ATI grading (τ_c = 0.65; 95% CI 0.54–0.76; *p* < 0.001), as shown in Table 3. The weighted kappa statistic was κ_w_ = 0.57 (95% CI: 0.46–0.68).

**Table 3:**
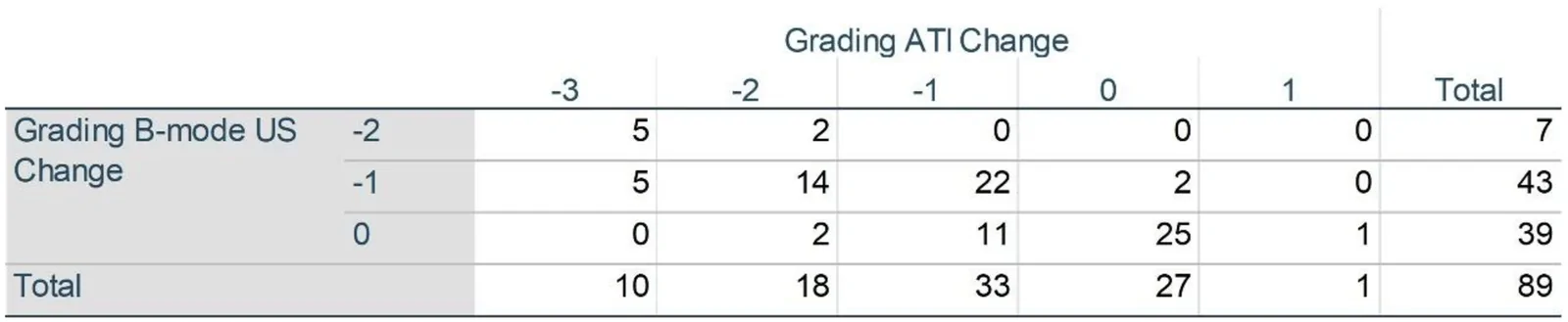
Association of changes in hepatic steatosis from baseline to early follow-up between B-mode US and ATI

**Fig. 8:**
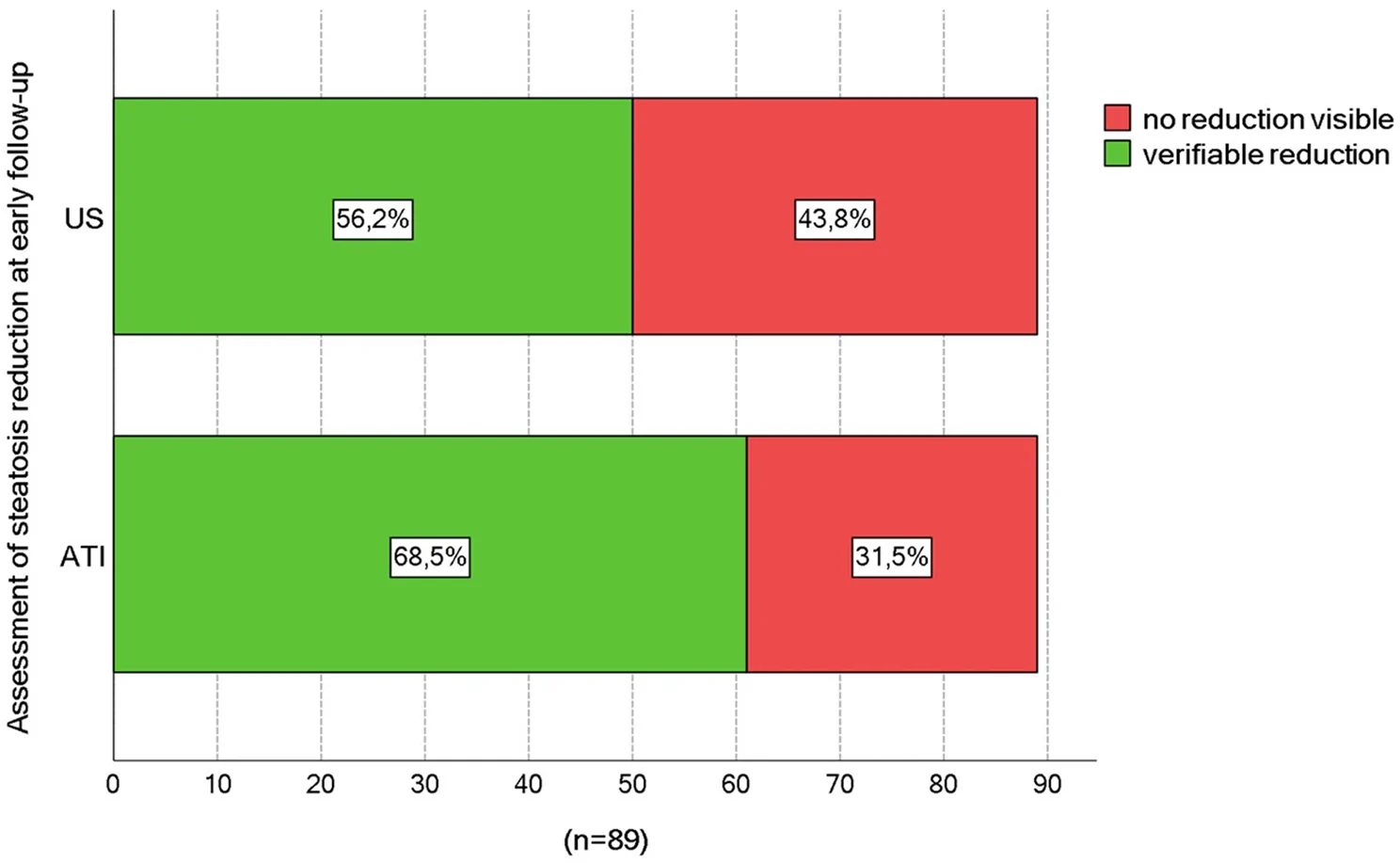
Proportion of patients with steatosis reduction at early follow-up in B-mode US and ATI grading

As illustrated in Fig. 8, a reduction in steatosis grading from baseline to early follow-up was more frequently detected using ATI (68.5%) than B-mode US (56.2%). According to the four-level grading system proposed by Hobeika et al., hepatic steatosis improved in 68.5% of patients based on ATI grading. Among patients without improvement, 19 had already been classified as aS0 at baseline. Among patients classified as ≥aS1, a reduction was observed in all but six patients. In the subgroup with only a 6-month follow-up, 72.7% (16/22) showed a reduction in ATI grading.

When absolute ATI values were considered, a decrease was observed in 94.4%. In the subgroup with a 6-month follow-up only, a reduction in ATI values was observed in 100% of patients.

## Discussion

Noninvasive and cost-effective diagnostic methods for hepatic steatosis are essential to address the increasing global burden of steatotic liver disease and its associated risk of progression to MASH and liver-related morbidity. Bariatric surgery has been shown to induce substantial regression or even complete resolution of MASLD [6, 7].

Rapid changes in hepatic fat content are best evaluated using short-term follow-up intervals. However, repeated assessment using liver biopsy or MRI is limited by invasiveness, high cost, and restricted feasibility in routine clinical practice [3]. A meta-analysis demonstrated that B-mode US provides diagnostic accuracy comparable to CT or MRI for detecting moderate-to-severe hepatic steatosis when histology serves as the reference standard [11].

More recently, ATI has emerged as a promising noninvasive quantitative US marker for liver fat assessment in MASLD [21], particularly in longitudinal follow-up studies with moderate to severe steatosis [27]. Moreover, ATI appears to outperform CAP in differentiating steatosis grades when MRI-PDFF is used as the reference standard [14].

In the present study, perioperative B-mode US combined with ATI demonstrated significant postoperative changes in 89 patients undergoing bariatric surgery. Both B-mode US findings and ATI values decreased significantly during early follow-up, in parallel with BMI reduction. These findings are consistent with previous studies reporting histological regression of MASLD following bariatric surgery [6, 7]. However, in line with previous studies [28], BMI was not significantly associated with ATI values, likely due to the limited variability in BMI within our cohort.

When absolute ATI values were analyzed, a reduction was observed in 94.4% of patients, suggesting that ATI is highly sensitive to early postoperative changes in hepatic fat content following substantial weight loss.

According to the grading system proposed by Hobeika et al. [24], hepatic steatosis grade improved in 68.5% of patients based on ATI, whereas only 56.2% showed improvement on B-mode US. These findings suggest that ATI may be more sensitive to subtle longitudinal changes in hepatic fat content than conventional B-mode US, however this needs to be proven in comparison with a gold standard method.

Using the Hobeika classification, hepatic steatosis was detected at baseline in 78.7% of patients by ATI compared with 88.7% using B-mode US. This differs from previous studies reporting higher detection rates with ATI than with B-mode US [23]. One possible explanation is the relatively high cutoff (>0.66) used to define aS1 steatosis. Previous studies have used lower thresholds, such as >0.64 [29]. Applying this lower threshold would have classified 90.0% of our cohort as having hepatic steatosis.

In parallel with BMI reduction, which was most pronounced within the first 3 months after surgery, ATI values also decreased more markedly during this interval than between 3 and 6 months. This supports the notion that hepatic fat reduction can be reliably detected by ATI at early postoperative time points. This observation is consistent with previous reports demonstrating that liver fat content can change within months [3].

Notably, all patients in the subgroup with only 6-month follow-up demonstrated reduced ATI values. This further suggests that ATI is highly sensitive to quantitative changes in hepatic steatosis even over relatively short time intervals, and that follow-up US at 6 months may reveal more pronounced changes than earlier assessments.

The correlation between B-mode US and ATI grading was strong both at baseline and during follow-up. Nevertheless, ATI detected steatosis regression more frequently, indicating that ATI provides clinically relevant complementary information to conventional B-mode US - particularly in follow-up examinations - by enabling the detection of more subtle changes over time. Since a gold-standard reference was not used in this study, diagnostic superiority of ATI over B mode US cannot be proven.

Nevertheless, the quantitative nature of ATI represents a major advantage over semiquantitative B-mode US because it provides objective measurements that are less dependent on examiner subjectivity. ATI may therefore reduce the inter- and intraobserver variability associated with visual steatosis grading. Interobserver agreement was not assessed in this study because previous investigations have already demonstrated excellent reproducibility [20].

Another notable finding was the rapid and significant postoperative reduction in liver size observed on B-mode US, which has not yet been systematically investigated in the literature. However, reductions in liver volume after bariatric surgery have previously been described using MRI and CT [8, 30]. Similar to ATI, the greatest reduction occurred within the first 3 months, with a smaller additional reduction between 3 and 6 months.

A limitation of this study is its single-center design and the absence of histological or MRI-based reference standards. However, routine MRI follow-up is impractical because of cost and limited availability, whereas liver biopsy is invasive and samples only a small portion of hepatic tissue. Consequently, biopsy is neither feasible nor ethically justified for serial liver fat quantification in large MASLD cohorts [3]. Furthermore, the pooling of two different examination time points into a single “early follow-up”, the heterogeneous follow-up intervals, the lack of reproducibility analysis, and the limited generalizability of the findings to other US platforms may have introduced bias and affected the external validity of the results. Strengths of this study include its prospective design, longitudinal assessment, and examinations performed by highly experienced sonographers.

## Conclusions

Early regression of hepatic steatosis following significant weight loss can be assessed by US, as evidenced by improvements in B-mode findings, ATI values, and liver size. ATI provides a quantitative assessment of hepatic fat content and may facilitate the detection of subtle longitudinal changes. Although the present study did not include an external reference standard such as MRI-PDFF, CAP, or histology, the observed reduction in ATI values was consistent with the expected improvement in hepatic steatosis observed on B-mode US following bariatric surgery. These findings suggest that ATI may be a useful tool for longitudinal monitoring of MAFLD, although further validation against established reference standards in larger prospective cohorts is required.

## Data Availability

The datasets generated and analysed during the current study are available from the corresponding author on reasonable request.
